# Pulmonary benign metastasizing leiomyoma in patients aged 45 years and younger: clinical features and novelty in treatment

**DOI:** 10.1186/s12890-023-02406-7

**Published:** 2023-05-15

**Authors:** Hao Su, Rong Fan, Hua Yang, Yan You, Lan Zhu, Fengzhi Feng

**Affiliations:** 1 Department of Obstetrics and Gynecology, Peking Union Medical College Hospital, National Clinical Research Center for Obstetric & Gynecologic Diseases, Chinese Academy of Medical Sciences & Peking Union Medical College, No.1 Shuai Fu Yuan, Dong Cheng District, Beijing, China; 2 Department of Pathology, Peking Union Medical College Hospital, Chinese Academy of Medical Sciences & Peking Union Medical College, Beijing, China

**Keywords:** Pulmonary benign metastasizing leiomyoma, Young age, Ovarian function, Treatment, Sirolimus, mTOR inhibitors

## Abstract

**Background:**

Pulmonary benign metastasizing leiomyoma (PBML) is the most common extrauterine spread of uterine leiomyoma, and its biological behavior is traditionally thought to be hormone dependent. Studies on older PBML patients have been previously reported, but limited literature has been published regarding the clinical features and treatment of PBML in young women.

**Methods:**

A total of 65 cases of PBML in women aged 45 years and younger were reviewed, including 56 cases selected from PubMed and 9 cases from our hospital. The clinical characteristics and management of these patients were analyzed.

**Results:**

The median age of all the patients at diagnosis was 39.0 years. PBML most commonly presented as bilateral solid lesions (60.9%), with other rare imaging manifestations. The median interval time from a pertinent gynecologic procedure to diagnosis was 6.0 years. A total of 16.7% of patients received careful observation, and all achieved stable status in a median follow-up time of 18.0 months. A total of 71.4% of patients were administered anti-estrogen therapies, including surgical castration (33.3%), gonadotropin-releasing hormone analog (23.8%) and anti-estrogen drugs (14.3%). Eight of 42 patients underwent surgical resection of metastatic lesions. Patients who underwent curative surgery for the removal of pulmonary lesions combined with adjuvant anti-estrogen therapies had favorable outcomes compared with those who only underwent surgical resection. The disease control rates of surgical castration, gonadotropin-releasing hormone analog, and anti-estrogen drugs were 85.7%, 90.0%, and 50.0%, respectively. For two patients, sirolimus (rapamycin) achieved successful relief of symptoms and control of pulmonary lesions without lowering hormone levels and causing estrogen deficiency symptoms.

**Conclusions:**

In the absence of standard treatment guidelines for PBML, maintaining a low-estrogen environment using different kinds of antiestrogen therapies has been the mainstream strategy and has satisfying curative effects. A wait-and-see strategy might be an option, but therapeutic approaches must be contemplated when complications or symptoms progress. For PBML in young women, the negative effect on ovarian function of anti-estrogen treatment, especially surgical castration, should be considered. Sirolimus might be a new treatment option for young PBML patients, especially for those who want to preserve ovarian function.

## Background

Benign metastasizing leiomyoma (BML) is a rare tumor that is characterized by benign leiomyoma located at any site in the body, such as the lung, abdominal cavity, retroperitoneum, lymph node, muscular tissue, or heart [1]. Lungs are the most frequent site of metastasis, and pulmonary BML (PBML) can present with solitary or multiple and unilateral or bilateral lung lesions, which may be misdiagnosed as metastatic cancer. Due to its rarity, diagnosis is usually made with a combination of medical history, imaging features and pathologic examinations. Although there is no standard treatment for PBML, several treatment options, including careful observation, surgical resection of the metastatic lesions, anti-estrogen therapies such as surgical castration and gonadotropin-releasing hormone analog (GnRHa), even chemotherapy and other targeted therapies, have been reported.

In two systematic reviews, the mean ages at diagnosis of PBML were 46.7 and 47.4 years, respectively, which are both within the late childbearing period [2, 3]. As a tumor traditionally thought to be hormone dependent, the clinical course of PBML is closely associated with estrogen levels. Therefore, similar to uterine leiomyoma (UL), young patients with PBML should be managed differently from older patients. To thoroughly describe the clinical characteristics among young women (age ≤ 45 years old) diagnosed with PBML, investigate the role and efficacy of various therapies and propose management strategies for this population, we comprehensively reviewed 9 cases of PBML in our hospital and 56 cases documented in the literature from PubMed. A promising new treatment, sirolimus (rapamycin), a mammalian target of rapamycin (mTOR) inhibitor, was also reported for the first time.

## Methods

**Fig. 1:**
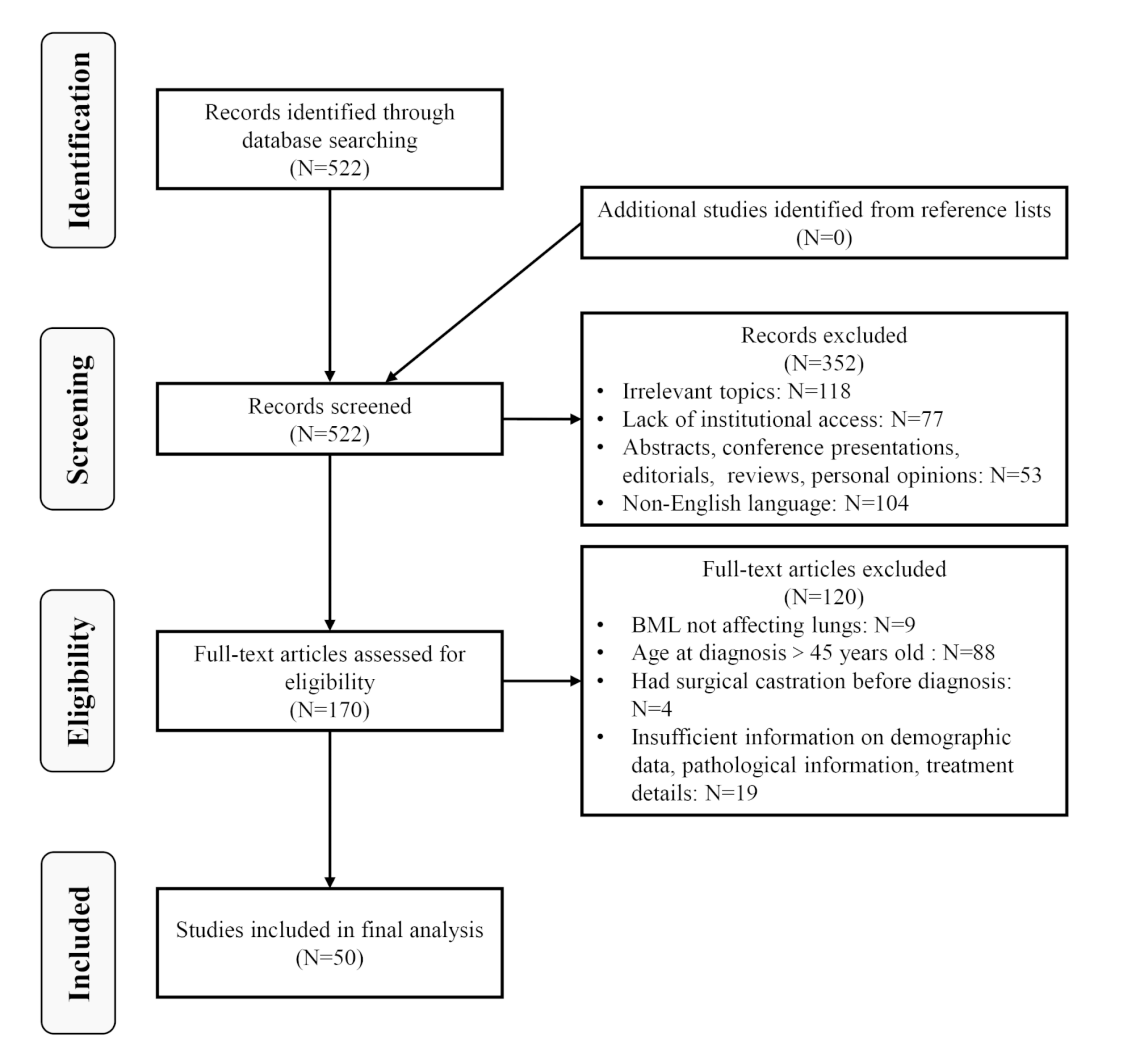
Flow chart for the process of article screening and selection on PubMed

A primary literature review was performed using the PubMed database from inception to 31 December 2021, using the following keywords to select for studies: “pulmonary benign metastasizing leiomyoma”, “benign metastasizing leiomyoma of lung”, “pulmonary benign leiomyoma”, and “benign leiomyoma of lung”. Additionally, we checked the included articles for references and conducted a citation screen. Articles were excluded if they were not published in English, did not include histological confirmation information, involved patients who were > 45 years old at diagnosis, did not discuss BML affecting lungs, or included patients with surgical castration before the diagnosis of PBML. After exclusion by reviewing titles, abstracts, and full-text articles, 50 studies consisting of 56 cases were selected to be reviewed (the detailed inclusion process can be found in Fig. 1). The retrospective analysis of patients with PBML diagnosed and treated in our hospital was approved by the Ethics Committee of Peking Union Medical College Hospital. We identified 9 PBML cases from January 2001 to November 2021 using the above inclusion criteria, among which 2 patients were previously enrolled in a clinical trial on the efficacy and safety of the mTOR inhibitor sirolimus in the treatment of symptomatic UL and leiomyomatosis (NCT03500367), and their written informed consent was obtained. All 9 patients’ diagnoses and treatments were discussed and determined by a multidisciplinary team (MDT) consisting of the Department of Obstetrics & Gynecology, Department of Pulmonary and Critical Care Medicine, Department of Thoracic Surgery, Department of Medical Imaging and Department of Pathology. Relevant permissions were acquired to access the medical records of these 9 patients under the authority of the same ethics committee. A database was generated and analyzed, including clinical characteristics, age at diagnosis, surgical procedure for diagnosis, imaging findings, other sites of metastatic disease, gynecologic history for UL, treatment options, and outcomes of follow-up, from these 65 cases.

The pulmonary lesion response to therapy of 65 cases from PubMed and our hospital were compared among three classifications, including radiologically progressive, radiologically stable and radiologically regressive. As follow-up information in the literature was less detailed, the outcomes of cases from PubMed were determined by a literature report. Two investigators completed the therapeutic effect judgment in the literature independently. When there was a divergence of opinion, we discussed this disagreement and settled it by consensus. Patient response to therapy in our hospital was evaluated by Response Evaluation Criteria in Solid Tumors 1.1 (RECIST 1.1) [4].

For data analysis, relevant data regarding the characteristics to be analyzed unfortunately were only available for certain cases. Thus, the number of patients who had a specific outcome suitable for analysis was reported. Descriptive statistics regarding patients’ characteristics were calculated. Continuous variables are described by means ± standard deviations (range) if they were normally distributed or as medians and interquartile ranges (IQRs) if they were abnormally distributed. Categorical variables are expressed by using counts (percentages).

## Results

The clinical characteristics of 65 patients with PBML are recorded in Table 1. The median age at diagnosis was 39.0 (IQR 36.0-43.3) years. Information on symptoms and radiographic findings was available in 62 and 64 patients, respectively. Thirty-four patients (54.8%) were diagnosed with PBML due to respiratory or other symptoms. Some patients had more than one symptom. Bilateral solid lesions were found in the majority of patients (N = 39, 60.9%). Extrapulmonary metastasis was reported in 15 patients (23.1%), in whom the abdominal or pelvic cavity (N = 13, 86.7%) was the most involved site. Fifty-one patients (78.5%) had undergone at least one surgical procedure for UL prior to the diagnosis of PBML, with a median time to diagnosis from gynecologic surgery of 6.0 (range 0.08-14.0) years.

**Table 1:** Clinical characteristics of PBML in women aged 45 years and younger

Variable	N	%		Variable	N
**Age (year)**	N = 60			**Symptom**	N = 62
Median 39.0				Asymptomatic	28
IQRs 36.0-43.3				Symptomatic	34
**Radiographic finding**	N = 64			Dyspnea	20
Bilateral solid lesions	39	60.9		Cough	10
Unilateral solid lesions	6	9.4		Chest pain/tightness	2
Solitary lesion	2	3.1		Pneumothorax	5
Miliary pattern	10	15.6		Hemoptysis	3
Cystic lesions	4	6.3		Backache	1
Interstitial lung disease	3	4.7		**Extrapulmonary metastatic sites**	N = 15
**Prior gynecologic surgery for UL**	N = 65			Abdominal/pelvic cavity	13
No	14	21.5		Retroperitoneum	4
1	41	63.1		Peritoneum	2
2	8	12.3		Omentum	2
3	2	3.1		unspecified	5
**Gynecologic procedure before diagnosis of PBML**	N = 51			Skeletal muscle	3
Hysterectomy	27	52.9		Mediastinum	2
Myomectomy	30	58.8		Bone	2
Hysteroscopy	1	2.0		Breast	1
**Time to diagnosis from gynecologic surgery (year)**	N = 47			Heart	1
Mean 6.3 ± 3.8					
Range 0.08-14.0 Median 6.0					

Diagnostic approaches were reported in 58 of the 65 cases, including CT-guided biopsy in 12 patients, transbronchial biopsy in 4 patients, and thoracoscopic surgery or thoracotomy in 42 patients. Before diagnostic procedures, 19 patients were reported to undergo preoperative lung function tests, 8 (42.1%) of which were normal and 11 (57.9%) of which were abnormal, including 5 with a reduction in diffusing capacity, 3 with decreased lung volumes (indicating restrictive lung disease) and 3 with unspecified abnormalities. Notably, patients with abnormal lung function all demonstrated bilateral pulmonary lesions, including 5 with a miliary pattern, 4 with bilateral solid lesions, 1 with bilateral cystic lesions and 1 with interstitial lung disease. In the patients with pathologic results, only one patient demonstrated local necrosis of a nodule in the right lower lobe, while the rest all lacked mitosis and necrosis. The positivity rates for smooth muscle antigen (SMA), estrogen receptor (ER), and progesterone receptor (PR) were 100% (N = 39), 97.7% (N = 43) and 100% (N = 40), respectively. The Ki-67 index of the tumors was low (N = 29, from < 0.5 to 5%).

Definite treatment, follow-up time and treatment-related outcomes were reported in 42 patients, including 33 cases from the literature and 9 cases from our facility. Careful observation was performed in 7 patients (16.7%) after the initial diagnosis. Most of the reported patients (N = 30, 71.4%) were administered anti-estrogen therapies, including surgical castration (N = 14, 33.3%), GnRHa (N = 10, 23.8%), and anti-estrogen drugs (N = 6, 14.3%, including aromatase inhibitors, progesterone and mifepristone). Surgical resection of the metastatic lesions for curative intent was performed in only 8 patients, among which 3 patients (7.1%) underwent surgical resection alone, 4 patients were treated combined with surgical castration, and 1 patient was treated combined with anti-estrogen drugs. Only 4 cases reported specified surgical methods, including 2 with wedge resection, 1 with lobectomy and wedge resection and 1 with parenchyma-sparing cautery resection and wedge resection. All of the patients who underwent surgery had a single or limited number of nodules in the lung, 7 of whom achieved radicality with all pulmonary lesions resected and only 1 of whom had a small amount of unresectable residual tumor adherent to major hilar vascular structures. However, none of the patients were reported to have undergone a preoperative lung function test, and only 1 patient had a postoperative lung function test. This patient, who had a limited number of bilateral solid pulmonary lesions and underwent parenchyma-sparing cautery resection and wedge resection, underwent a total of 7 surgeries to remove all the pulmonary lesions, and her respiratory function test performed after the last surgery showed near-normal results (forced vital capacity (FVC): 77%, forced expiratory volume in 1s (FEV1): 64%, FEV1/FVC: 0.83). The follow-up time and outcomes of the above conventional treatment options are summarized in Table 2.

**Table 2:** Follow-up and outcomes of conventional treatment for PBML patients (N = 40) from literature and our hospital

Treatment options	No. of patients	Follow-up time (month)	Outcomes (N, %)		
			Radiologically progressive	Radiologically stable	Radiologically regressive
Observation	7	Median 18.0 IQRs 12.0–38.0	0 (0%)	7 (100.0%)	0 (0%)
GnRHa	10	Median 12.0 IQRs 8.5–20.3	1 (10.0%)	5 (50.0%)	4 (40.0%)
Surgical castration^*^	14	Median 19.5 IQRs 11.3–29.5	2 (14.3%)	9 (64.3%)	3 (21.4%)
Anti-estrogen drugs^†^	6	Median 15.5 IQRs 3.5–33.5	3 (50.0%)	3 (50.0%)	0 (0%)
Surgery^††^	3	Median 18.0 IQRs 15.0-20.6	2 (66.7%)	0 (0%)	1 (33.3%)

^*^including 4 patients simultaneously treated with surgical resection of pulmonary metastatic lesions, and all of them achieved no recurrence; ^†^including 1 patient simultaneously treated with surgical resection of pulmonary metastatic lesions, and she achieved no recurrence; ^††^patients only received surgical resection of pulmonary metastatic lesions

**Fig. 2:**
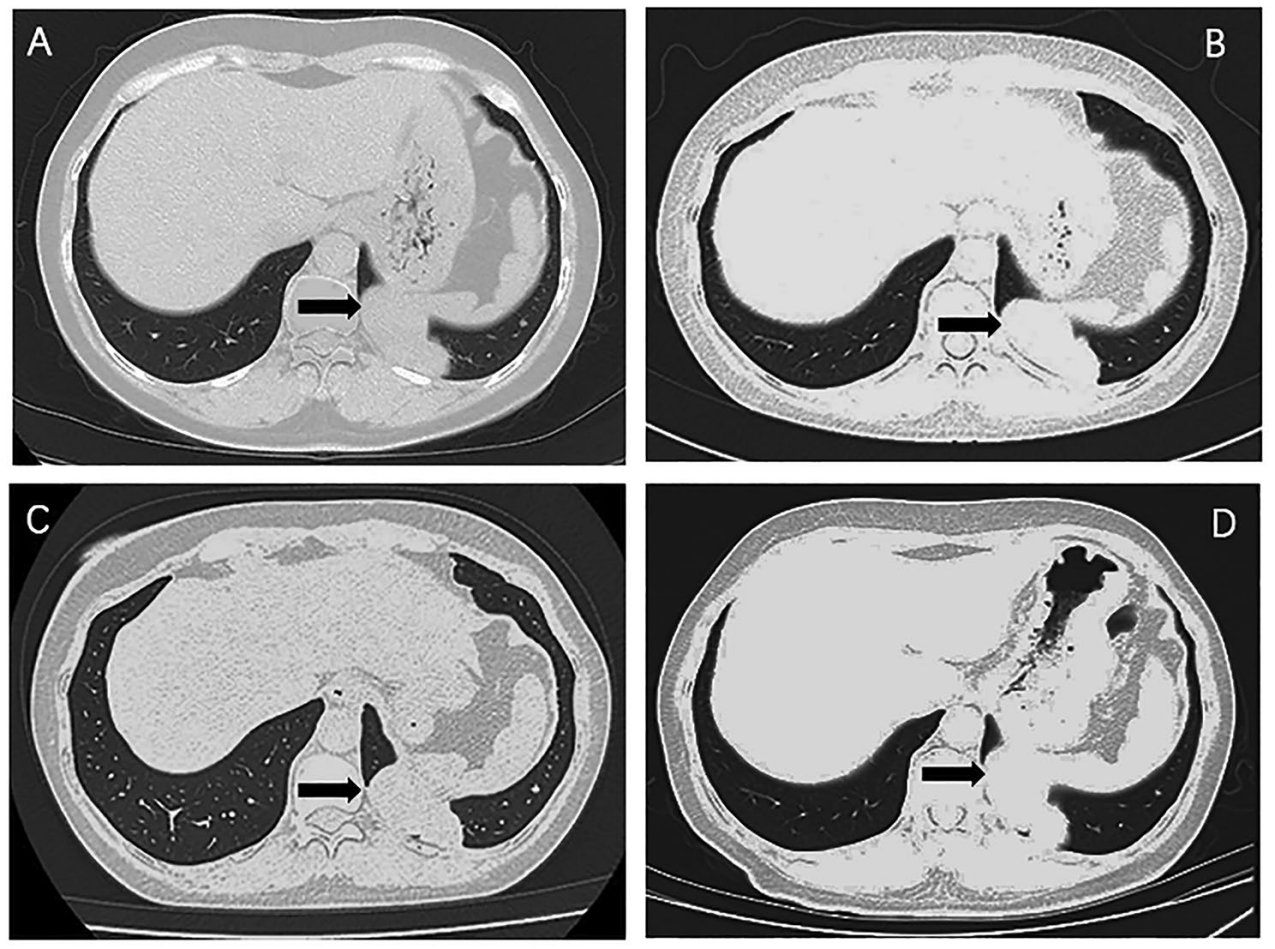
Changes of the mass in the left lower lobe (black arrow) of Case 1 during sirolimus treatment A. 5.4 × 2.6 cm before treatment; B. 6.2 × 2.4 cm at 6 months; C. 6.2 × 2.6 cm at 18 months; D. 6.2 × 2.6 cm at 30 months

Moreover, 2 patients (4.8%, referred to as Case 1 and Case 2) in our hospital were administered sirolimus instead of conventional therapies. Case 1 participated in March 2019, and sirolimus 2 mg/day (adjusted according to blood concentration of rapamycin) was administered. The mass in the left lower lobe was 5.4 × 2.6 cm in size before treatment. At 6 months of treatment, chest CT found that the mass, sized 6.2 × 2.4 cm, was slightly larger, and other bilateral lung nodules did not change. Considering that the patient’s cough had been relieved since taking the drug, the treatment was continued. At 18 and 30 months of therapy, her cough completely disappeared, and chest CT showed a mass in the left lower lobe (sized 6.2 × 2.6 cm at both visits) that was stable (Fig. 2) according to RECIST 1.1, with other nodules remaining unchanged. Moreover, we performed whole-exome sequencing (WES) of her lung and uterine tumors. As a result, 9 common mutant genes, including SLFN11, PPP1R12B, CNOT1, RNPC3, SLC23A2, OTOF, MTMR14, OR2T24, and AHNAK, were found.

**Fig. 3:**
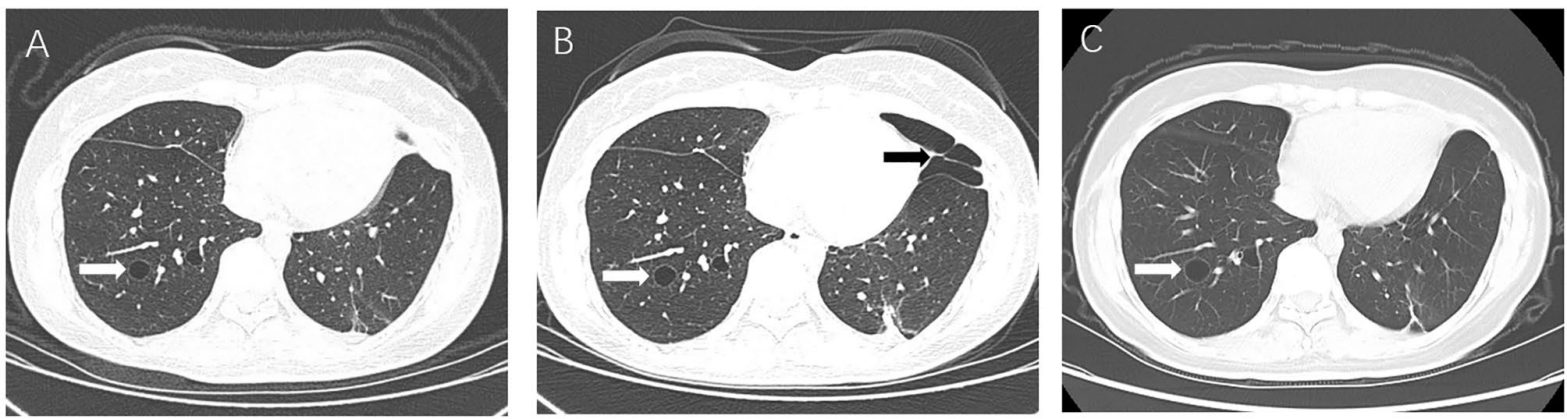
Changes in the pulmonary lesions of Case 2 during sirolimus treatment The lesions (white arrow) remained stable during 12 months of sirolimus treatment (from Fig. 3A and C). The asymptomatic pneumothorax (black arrow in Fig. 3B) at 6 months totally disappeared at 12 months of sirolimus treatment (Fig. 3C).

Case 2 previously experienced pneumothorax onset at an average frequency of 2 months before sirolimus treatment and had to undergo multiple bullectomies to relieve her obvious dyspnea. In November 2020, she presented to our hospital and started taking sirolimus 2 mg/day. At 6 months of follow-up, she had a slight but asymptomatic pneumothorax found by chest CT. She did not receive any treatment other than sirolimus. After 12 months of therapy, chest CT showed that the pneumothorax at 6 months completely disappeared, and other cystic lesions were stable (Fig. 3).

After starting the treatment, Case 1 developed mouth ulcers and oligomenorrhea, while Case 2 had facial acne. Case 1’s mouth ulcer gradually ameliorated without intervention, but her oligomenorrhea remained. Hematologic, renal, and liver function tests were notable only for a mildly increased triglyceride level. Hormonal parameters, including luteinizing hormone, follicle-stimulating hormone, estrogen, progesterone, testosterone, and prolactin, were all within the normal range, and the patients did not report any low-estrogen-related symptoms. All the adverse events mentioned above were grade 1–2 according to the Common Terminology Criteria for Adverse Events, and no grade 3–5 adverse events were observed during follow-up [5].

## Discussion

Since it was first recognized by Steiner in 1939, most of the articles about PBML have been case reports [6]. The only two current systematic reviews have focused on BML and PBML of all ages and had fewer details about their treatment options. To the best of our knowledge, this is the first literature review of young patients (age ≤ 45 years old) with PBML and the third largest series of patients with PBML in a single facility. Miller et al. reported an older group (mean age at diagnosis was 54.1 years) of 10 PBML cases [2]. In their case series, only 30% of the patients presented with symptoms, while the percentage in our database was 54.8%. Apart from the most common bilateral solid lesions, the miliary pattern accounts for the largest proportion of rare imaging manifestations, which was not reported in Miller’s series. Solitary lesions, which occur in 13% of PBML patients of all ages, were only reported in 3.1% of young cases [7]. Almost 80% of young women with PBML had prior uterine procedures. The definite diagnosis of PBML relies on pathology. Adequate tissue should be acquired by different techniques, including CT-guided biopsy, thoracoscopic surgery and thoracotomy. All specimens needed to be analyzed for SMA, ER, PR and Ki-67 to differentiate them from other pulmonary primary or secondary tumors, such as sarcoma or carcinoma. What needs to be emphasized is that PBML is a rare disease, and an MDT including different disciplines should be involved to establish the diagnosis based on medical history, imaging and pathologic features and finally decide the subsequent treatment options.

There are several treatment options for PBML. The first choice is excision by surgery to remove the foci. According to the results of lung function tests in our study, a great proportion of PBML patients, owing to the most common radiological manifestation of bilateral pulmonary lesions, had diffusing capacity alterations or restrictive lung disease, although this disease is always supposed to have indolent biological behavior, and there have even been reports of patients developing respiratory failure [8]. Thus, it is necessary to test lung function with spirometry before surgery to better evaluate whether the patient could be a candidate for lung surgery. The impact on lung function after pulmonary surgical resection should also be taken into consideration. Repeat metastasectomy to remove as many lesions as possible still needs to be wielded with caution, and appropriate surgical methods (such as parenchyma-sparing cautery resection) should be selected to preserve lung function [9]. Our study results showed that for young patients with a single or limited number of lung lesions, curative surgery for the removal of pulmonary lesions combined with adjuvant anti-estrogen therapies is important for achieving a favorable outcome. Interestingly, 70% of the patients in Miller’s series (age at diagnosis mainly in peri- and post-menopause) received no additional therapy after surgical resection, and they all had stable disease or an asymptomatic slight increase in the residual lesions with a median follow-up of 12 years [2]. This is probably because women with PBML at a young age have a longer disease course and higher level of sex hormones compared with the older group.

Patients treated with careful observation achieved a good outcome in our database but with a relatively short follow-up time. For young patients with long-term follow-up, its role still needs further verification.

The therapeutic effect of chemotherapy on PBML is controversial and debatable. According to 2 reported cases, chemotherapy has shown no treatment effect due to a lack of signs of necrosis, thrombosis, or fibrosis on pathologic examination of tumors resected after chemotherapy [9, 10]. In contrast, another case series reported several chemotherapy schemes (such as high-dose ifosfamide, ifosfamide + dacarbazine, gemcitaine and oral etoposide) that achieved stable, partial remission and even complete remission in treating PBML [11]. Histologically, BML tumors are not very mitotically active. Thus, cytotoxic treatment is theoretically supposed to be ineffective. Furthermore, using chemotherapy with significant adverse effects to treat such a benign disease is not preferential in clinical practice.

As previous research considered that BML growth was dependent on estrogen stimulation, anti-estrogen therapy has been used as a first-line option in the treatment of PBML. More than 70% of the patients from the database received anti-estrogen therapy. Anti-estrogen drugs used in the treatment of PBML, including aromatase inhibitors, progesterone and mifepristone, seem to have mixed positive and negative effects in young women, with apparently varying therapeutic outcomes in both our study and other studies [12]. Surgical castration and GnRHa are the most frequently used treatments and show a more favorable effect on disease control. However, the side effects of estrogen deficiency will reduce quality of life and even increase morbidity and mortality [13]. Additionally, a considerable proportion of young patients in our study only achieved stable disease after undergoing surgical or chemical castration. Therefore, for young women with PBML, the pros and cons of such treatment options should be weighed between the impact on the women’s reproductive system and the therapeutic effect on disease. Importantly, some patients with anti-estrogen manipulation demonstrated disease progression in our study and in other studies, which suggests that there may be biological mechanisms of PBML other than hormones [14].

We report two cases of sirolimus in treating PBML for the first time. Studies have shown that mTOR inhibitors are a potential effective treatment for UL and other special types of leiomyomas, including PBML. The mTOR pathway is one of the most highly upregulated pathways in UL and is necessary for estrogen-dependent cell growth in UL [15, 16]. The rapamycin analog WAY-129,327 inhibited mTOR signaling and shrank tumors in an Eker rat animal model [17]. In another disease also caused by activation of the mTOR signaling pathway and characterized by smooth muscle cell infiltration, lymphangioleiomyomatosis, sirolimus has shown persistent efficacy and long-term safety [18]. Furthermore, Zhang et al. recently reported the first successful control of disease with sirolimus in treating intravenous leiomyomatosis [19], and another mTOR inhibitor, everolimus, in treating a PBML patient achieved partial remission lasting 57 months [11]. In Case 1, we also obtained WES data from PBML and UL samples. Among the 9 common mutant genes, we speculated that the mutation of SLFN11 might cause dysfunction of the mTOR pathway. A study has shown that SLFN11 can act as a tumor suppressor by suppressing the mTOR pathway by targeting RPS4X in hepatocellular carcinoma, which suggests that its mutation may lead to the loss of its inhibitory effect on mTOR [20]. This indicates that activation of the mTOR pathway may be the chief reason for PBML occurrence in Case 1 and is in accordance with sirolimus’s therapeutic effect in her case.

The main adverse effects of sirolimus include mouth ulcers, menstrual abnormalities, acne, and diarrhea. Laboratory abnormalities have been reported, including hyperlipidemia, hyperglycemia, anemia, thrombocytopenia, and liver enzyme elevation [21]. In Cases 1 and 2, we observed mouth ulcers, oligomenorrhea, acne and elevated triglyceride levels, but these were all mild and tolerable with 2 mg/day sirolimus. Furthermore, adverse events could decrease over time [18]. Notably, these patients’ ovarian function was normal during sirolimus treatment, and they did not experience any estrogen deficiency-related symptoms. This is different from conventional anti-estrogen therapies. Some studies have found that rapamycin may have a protective effect on the ovaries. Compared to the control group, rapamycin-treated rats had a 2-fold increase in the number of primordial follicles after 10 weeks of treatment, which indicated that rapamycin can maintain the ovarian reserve and prolong ovarian lifespan [22, 23].

The minimal treatment goal of PBML is to maintain the stability of disease, but the ideal outcome is disease regression. However, for those young women who took anti-estrogen therapy, especially surgical castration and GnRHa, but only achieved stable disease or even had disease progression, sirolimus is obviously a more appropriate treatment option for them, with less negative impact on their ovarian function.

Our study inspires the exploration of other targeted therapies. First, given that angiogenic factors, including vascular endothelial growth factor (VEGF), have been found to be important in the pathogenesis of leiomyoma [24], Chhabra et al. recently reported a prolonged response with bevacizumab in a patient with BML [25]. In this case, the anti-VEGF monoclonal antibody bevacizumab was initiated after traditional hormonal manipulation failed. After 3 years of treatment, the patient continued to have stable disease. This highlights the importance of anti-angiogenesis treatment (such as anti-VEGF therapy) as a treatment option for PBML. Second, multiple kinase-related signaling pathways also play a vital role in the development of leiomyoma. In one case, the multikinase inhibitor sorafenib as a treatment for PBML was reported to achieve stable disease lasting 6 months [11]. In summary, the application of targeted drugs in treating PBML provides a new prospective, but further validation is still warranted.

The main limitation of our study is that most of the data were extracted from case reports or case series. Selection bias could inevitably exist in these articles, and the information provided was occasionally brief, scarce, and not comprehensive. For instance, the pulmonary lesion response to therapy in the literature was estimated according to the articles’ description, rather than assessed by the objective standard RECIST 1.1 like patients from our hospital were, which might cause misjudgment of the therapeutic effect of different treatment options, even if we tried our best to accomplish this with two independent investigators. Additionally, in Cases 1 and 2, although no objective regression of the pulmonary lesions was observed, they were clinically improved. The possibility of a placebo effect cannot be excluded but is unlikely because symptoms had not recurred during follow-up. Moreover, in the first 6 months of sirolimus treatment, symptomatic relief but slight progression in imaging occurred in both Cases 1 and 2, which indicates that sirolimus for PBML may need some time to take effect. In the future, more research is needed to replicate these findings and further evaluate its safety.

## Conclusions

In conclusion, PBML is a rare disease characterized by benign leiomyoma existing in the lungs and should be individually treated according to the patients’ age, symptoms and imaging characteristics of pulmonary lesions. Patients’ hormonal status should be determined because it can direct the choice for hormonal blockade. For young women with PBML, it is essential to note the impact of anti-estrogen therapies, especially surgical castration, on ovarian function. Sirolimus is a potentially effective drug for young PBML patients, especially for those who want to preserve their ovarian function. In the future, gene sequencing should be applied to select patients better suited for sirolimus, and more clinical trials are needed.

## Data Availability

The data used and analyzed during the current study are available from the corresponding author on reasonable request.

## List of abbreviations

BML: benign metastasizing leiomyoma
PBML: pulmonary benign metastasizing leiomyoma
GnRHa: gonadotropin-releasing hormone analog
UL: uterine leiomyoma
mTOR: mammalian target of rapamycin
RECIST 1.1: Response Evaluation Criteria in Solid Tumors 1.1
MDT: multidisciplinary team
IQRs: interquartile ranges
SMA: smooth muscle antigen
ER: estrogen receptor
PR: progesterone receptor
FVC: forced vital capacity
FEV1: forced expiratory volume in 1s
WES: whole exon sequencing.
